# Posttraumatic chronic boutonniere deformity correction using ala carte reconstruction and local anesthesia surgery, A case report

**DOI:** 10.1016/j.ijscr.2024.110418

**Published:** 2024-10-05

**Authors:** Wildan Latief, Raden Handidwiono

**Affiliations:** Department of Orthopaedics and Traumatology, Faculty of Medicine, Universitas Indonesia – Cipto Mangunkusumo Hospital, Indonesia

**Keywords:** Boutonniere, Local anesthesia, Single stage, Chronic, Case report

## Abstract

**Introduction and importance:**

Posttraumatic boutonnière deformities are complex clinical problems that are often poorly understood. Nevertheless, there are no established therapy guidelines, and there is little data to support the various treatment outcomes. In this report, we want to report on the treatment using an ala carte approach of already established procedures.

**Case presentation:**

An 18-year-old male, complained about a crooked left middle finger for 1 year before admission, with a history of traumatic injury due to getting slashed by a machete. The operative procedure of releasing the central slip, lateral band, and transverse retinacular ligament, reconstruction using the Ohshio method, terminal tendon tenotomy, and fixation using K-wire. Intraoperative range of motion was evaluated. After 3 months post-operation, the patient was able to do full flexion and extension of the middle finger and after 1 year follow-up, the alignment and the function were satisfactory.

**Clinical discussion:**

Chronic boutonnière deformity occurs when central slip injury prevents full PIP joint extension, causing lateral slip tension and DIP extension. Acute cases benefit from splinting and rehabilitation to avoid permanent deformities. Splinting, including relative motion flexion splinting, is crucial early on. For chronic cases, surgery such as the Curtis procedure or central slip tenotomy may be necessary. The Curtis method involves staged tendon repair, while tenotomy focuses on direct tendon reconstruction. Both approaches show promising results but may leave residual lag. Individualized treatment and timely intervention are essential for optimal outcomes.

**Conclusion:**

The Ala carte approach of reconstruction procedure using anesthesia yields good results. The importance of an intraoperative active range of movement evaluation plays a crucial role so that correction can be made accordingly.

## Highlights

• The surgery involved releasing the central slip, lateral band, and transverse retinacular ligament (TRL) using the Ohshio method, followed by tenotomy and fixation with K-wire.
• Post-operative results showed full flexion and extension of the middle finger, with satisfactory alignment and function after a 1-year follow-up.
• The ala carte reconstruction approach under local anesthesia is effective for correcting chronic boutonnière deformity, emphasizing the importance of intraoperative ROM evaluation.

## 1 Introduction

Posttraumatic boutonnière deformities are complex clinical problems that are often poorly understood. A boutonnière deformity consists of flexion of the proximal interphalangeal (PIP) joint and hyperextension of the distal interphalangeal (DIP) joint [1]. Boutonniere deformity primarily arises from traumatic damage, although it can also result from non-traumatic factors such as rheumatoid arthritis, chronic flexion contracture, flexor pulley disturbances, and burns [1,2].

Chronic injuries of the tendons would have several complications that make it harder to manage, such as tendon retraction, tendon callus lengthening, scaring, adhesions, and joint contractures [3]. During surgical procedures to treat chronic boutonniere deformity, several surgeons have prioritized the damaged central slip and have sought to restore the central tendon anatomically using various techniques. Currently, several treatment approaches are being used, including conservative treatment, anatomical reconstruction, tendon grafting, tendon transplantation, central tendon shortening, and lateral band reconstruction. Nevertheless, there is a lack of a universally accepted treatment protocol and the available evidence on the different therapy results is inadequate [4,5]. The purpose of the case study was to provide a detailed account of the progression of boutonniere deformity and its treatment, as well as to present the clinical outcomes of corrective surgery performed utilizing an ala carte reconstruction technique and local anesthesia. This study has been reported in line with the SCARE criteria [6].

## 2 Case illustration

An 18-year-old male was complaining about a crooked left middle finger for 1 year before admission. One year ago, the patient's left middle finger was injured by a machete. The patient did not seek any medical treatment after the incident. After a few weeks, the patient felt the middle finger became crooked. The patient was right-hand dominant, and he had just graduated from high school.

One year later, the patient went to a regional hospital but was referred to a bigger hospital due to the complex nature of the deformity. The patient already tried physiotherapy and splinting in the previous hospital, but there was no improvement.

**Fig. 1:**
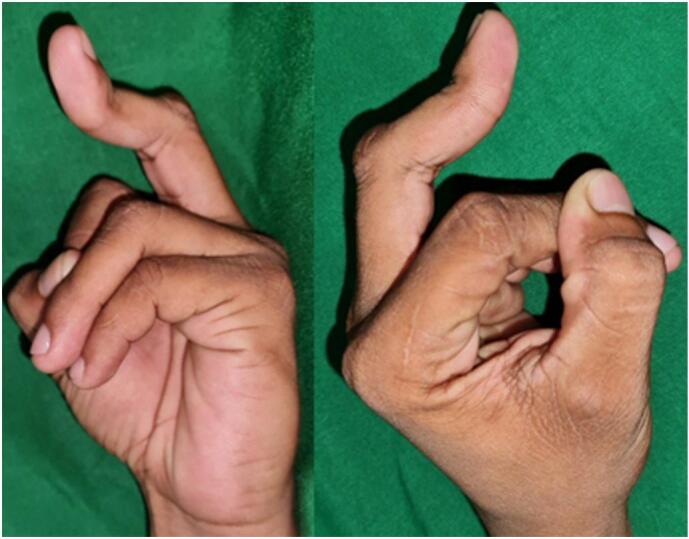
Clinical presentation of the left middle finger.

Examination of the hand showed the middle finger of the patient had flexion on the proximal interphalangeal (PIP) joint and slight hyperextension of the distal interphalangeal (DIP) joint, resembling the boutonniere deformity (Fig. 1). There was no wound or scar visible on the middle finger. On palpation, there was no pain or neurosensory problem, and capillary refilling time was less than two seconds. The movement of the other fingers was normal. The range of movement of the middle finger was 0–90 degrees at the metacarpophalangeal joint (MCP), 30–90 degrees at the proximal interphalangeal joint (PIP), and 30–40 degrees at the distal interphalangeal joint (DIP) both actively and passively.

**Fig. 2:**
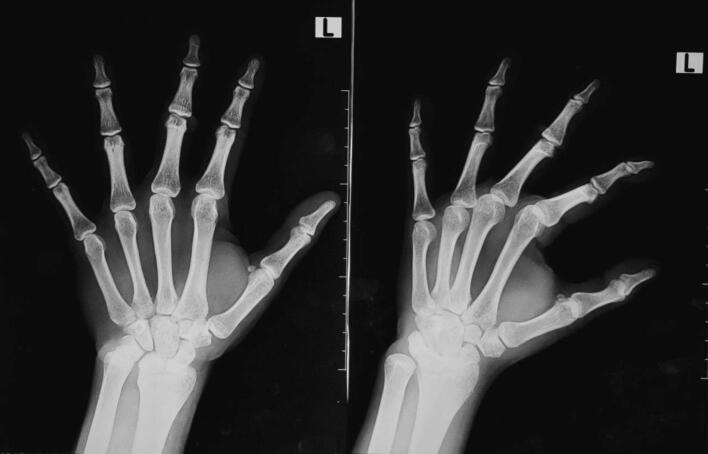
Pre-operative plain AP/Oblique X-ray of the left hand.

A standard AP and oblique plain radiograph of the hand was ordered in the previous hospital, showing a deformity on the DIP and PIP joint (Fig. 2). There were no signs of arthritis on the PIP and the DIP joint of the middle finger, such as narrowing joint space and sclerosis on the joints. The patient was diagnosed with Chronic boutonniere deformity of the left middle finger Burton classification type 2.

**Fig. 3:**
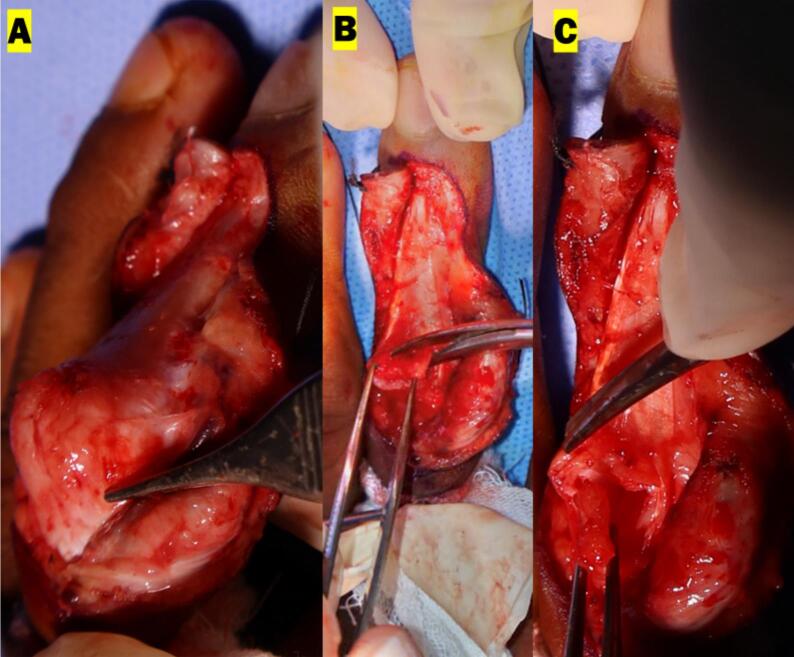
Intraoperative procedure for PIP joint: (A) Release central slip, lateral band, and TRL (B) Central slip tenotomy (C) TRL transection for Ohshio method.

**Fig. 4:**
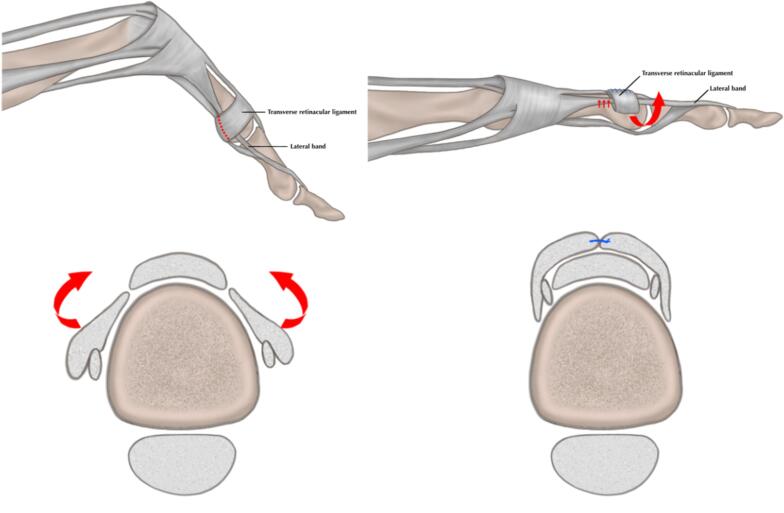
Image illustrates the Ohsio technique.

**Fig. 5:**
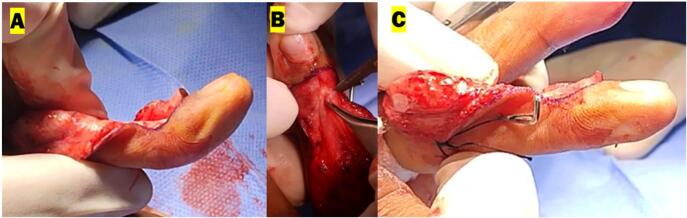
Intraoperative procedure for DIP joint: (A) Evaluation of active extension of DIP joint showed hyperextension (B) Terminal tendon tenotomy (C) Evaluation of active extension of DIP joint after terminal tendon tenotomy showed full extension without hyperextension.

The surgery intervention was fibrotic tissue released, central slip tenotomy and central slip reconstruction using the Ohshio method [7], and fixation using k-wire. The procedure was performed using local anesthesia with infiltration and finger block techniques. The initial cut was performed on the back side of the middle finger and continued until reaching the fibrotic tissue. The fibrotic release was performed around the central slip, the lateral band, and the TRL. After the release, the central slip tenotomy was performed and the PIP was evaluated. The extension of PIP still was not maximal. Both sides of the TRL were then released at the volar plate and flipped dorsally. The lateral band was moved dorsally from the PIP joint and the edge of the released TRL was sutured back dorsally holding the lateral band on the posterior side of the PIP joint. We evaluated the PIP joint extension intraoperatively and we found that the normal ROM was achieved. We proceed to fixate the PIP joint using a 0.8 K-wire diagonally. We moved to evaluate the DIP joint range of movement. When the patient was asked to extend the finger, hyperextension of the DIP joint was observed. A partial incision was made at the terminal tendon (terminal tendon tenotomy) of the extensor digitorum communist (EDC) to correct the hyperextension from the DIP joint (Fig. 3). After tenotomy the DIP joint extension intraoperatively was corrected, and no more hyperextension was observed. We then fixate the DIP joint using a 0.8 K-wire longitudinally. The wound then was washed with normal saline and the wound was closed using vertical mattrass suture (Fig. 4, Fig. 5).

**Fig. 6:**
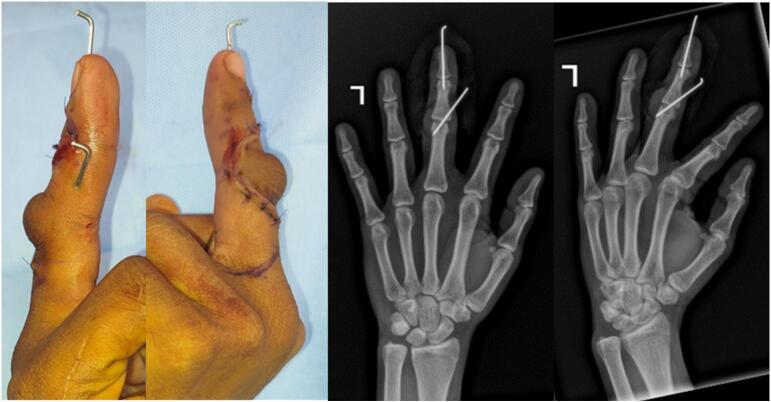
Post-operative condition.

The patient was discharged on the same day after the operation. The post-operative examination showed better alignment of the left middle finger. After approximately 4 weeks post-operation, the K-wire was removed, and the patient was encouraged to move the middle finger (Fig. 6).

**Fig. 7:**
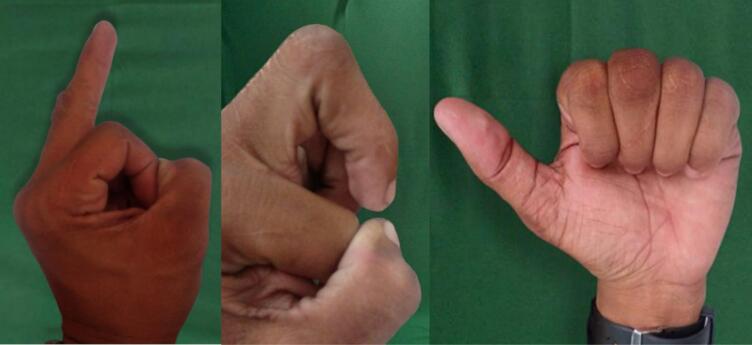
Follow up after 1 year post operation.

The patient was able to fully flex the finger on the PIP joint, while the DIP joint showed a reduction in active ROM although full flexion on passive ROM was achieved without any pain for 3 months post-operation. The patient was able to maintain the alignment and had no problem in grasping or doing daily activities. The patient also felt satisfied with the appearance and the function of the finger. One year after the follow-up, no complaint was felt by the patient, and the patient was able to do daily activities without any problem. No pain, no deformity, no limited range of movement was observed (Fig. 7).

## 3 Discussion

Chronic injury involving the central slip of the extensor mechanism results in boutonniere deformity [3]. Usually, it is the consequence of dorsal PIP laceration, PIP volar dislocation, or direct blow to the flexed PIP joint. After the injury, the patient would not be able to have a full active extension of the PIP joint due to flexion deformity and retraction of the extensor mechanism. The tension migrates to the lateral slips and gradually stretches the triangular ligament which results in the extension of the DIP joint [3]. Chronic boutonnière deformity was said to be more than 8 weeks post-injury. In this patient, we found the injury was caused by a machete one year before admission to the hospital.

Splinting and rehabilitation are crucial in the treatment of acute boutonniere deformity in the fingers. In the acute phase, splinting is essential to prevent further trauma that could result in permanent deformities [8]. Closed extension splinting is frequently the first choice for treating boutonniere deformity, as it facilitates the healing of the core slide [9]. Relative motion flexion splinting is effective in the early management of boutonniere deformities, enabling immediate active motion and hand use following injury or repair [10]. This technique positions the affected finger in a slightly bent position in relation to the neighboring fingers. It is suitable for treating injuries to the flexor tendons or early cases of boutonniere abnormalities [11]. Rehabilitation is also a key component in managing boutonniere deformity. Conservative treatment typically involves the use of dynamic splints and hand therapy to improve outcomes [12]. In cases of acute and flexible boutonniere deformities, continuous splinting in extension for the PIP joint while allowing the distal interphalangeal joint to flex can help stretch out contracted lateral bands [13]. Additionally, rehabilitation following immobilization should include flexion at the distal interphalangeal joint to prevent the development of boutonniere deformity post-immobilization [14].

In this patient, splinting and rehabilitation were already attempted but did not yield significant results or outcomes. This may be attributed to the patient presenting to the orthopedic clinic late after the acute phase had passed. Splinting is a crucial component in the management of acute boutonniere deformity. Wu et al [15], found that splinting led to an 84 % success rate in treating sagittal band injuries and extensor tendon subluxation, with complete symptom resolution at 13 weeks. In the acute phase of boutonniere deformity, splinting is essential for stabilizing the injured joint and facilitating central slip healing. Merrit et al [10], also emphasized the importance of splinting in the early management of boutonniere deformities, such as relative motion flexion splinting, which enables immediate active motion and hand use post-injury or repair. Nevertheless, for chronic boutonniere deformities, splinting remains beneficial. Furthermore, in cases of permanent chronic boutonniere, a technique involving the use of serial casting, followed by flexion splinting with relative motion and hand use for a duration of 12 weeks, has been employed to improve outcomes [10].

Physical examination in chronic boutonnière deformity showed a positive Elson test to identify central slip injury. The Elson test was performed by flexing the PIP joint at 90 degrees and asking the patient to actively extend the DIP joint while giving some resistance to the PIP [16]. In this patient, the Elson test was positive for a rigid DIP joint and has a weak extension at the PIP joint with extension at the DIP.

In case of failed conservative treatment, several surgical interventions have been suggested for the treatment of boutonnière deformity. Unfortunately, a consensus has not yet been reached. The Curtis procedure is an effective technique for correcting the disparity between the extensor and flexor tendons in boutonnière deformity. Curtis outlined a methodical way to rectify this malformation. Stage I consists of the surgical procedure of tenolysis, which comprises the release of the extensor tendon. Stage II involves the division of the transverse retinacular ligament. Stage III entails the elongation of the lateral bands over the middle phalanx, whereas Stage IV focuses on the restoration of the central extensor tendon. The operation is terminated if the deformity is rectified at any point. Stage II is conducted when complete extension is not attained following Stage I. Stage III is conducted if, following Stage II, there is still a deficiency of less than 20 degrees in PIP extension. Stage IV is triggered when there is a deficiency of more than 20 degrees in PIP extension or when complete extension is not attained after Stage III [17,18].

A study by Khoo et al. reported on 18 patients who underwent the Curtis procedure, with 7 requiring Stage IV reconstruction. Nine patients (50 %) achieved full DIP extension. Among the 7 patients who required Stage IV reconstruction, near-normal DIP joint function was achieved, with an average residual lag of 10 degrees [18]. Similarly, Curtis et al. reported 23 patients treated with this technique, with 6 requiring Stage IV reconstruction. The 17 patients who only underwent Stages I-III lacked an average of 10 degrees postoperatively, whereas the 6 patients who required Stage IV improved but still had an average deficiency of 17 degrees postoperatively [17].

Another approach for boutonnière deformity correction involves central slip tenotomy. This method directly incises and reconstructs the central slip tendon. Several authors have reported different versions of this reconstructive technique. Lee et al. [5] reported a method to release the tissue between the lateral bands and the central slip, by using a bilateral incision and release. The first portion of the central slip tendon was meticulously repaired to the lower end utilizing a loop suture technique, along with additional reinforcement from a suture anchor. Pardini et al. [19] performed excision scar tissue from the central slip approximately 1 cm proximal to the base of the middle phalanx, followed by attempting a direct end-to-end repair and suturing the lateral bands to the central slip. Grundberg et al. [20] conducted a surgical procedure where he removed a 3 mm segment of the central slip and then repaired the central slip without affecting the lateral bands. Afterward, a K-wire was put into the PIP joint when it was fully extended to provide temporary stabilization. In a study conducted by Lee et al. involving 13 patients with trauma-induced boutonnière deformity. Post-operative showed significant improvement in PIP with an average residual lag of 21.9 degrees. According to Souter's criteria, 9 patients (69.2 %) reported good [5]. Meanwhile, Pardini et al. reported 27 digits with trauma-induced boutonnière deformity corrected using central slip tenotomy yielded 59 % good and 41 % poor outcomes based on Souter's criteria [19]. Grundberg et al. presented that 3 out of 7 patients achieved full PIP joint extension after the central slip tenotomy procedure [20].

Littler et al. also reported a technique to correct boutonnière deformity. This technique involves repairing the chronic boutonnière deformity by utilizing the lateral bands. This procedure involves the detachment of the lateral bands from the oblique retinacular ligament and then sutured to the insertion of the central slip to correct the deformity. Post-operatively K-wire was inserted at the PIP joint in full extension. This technique was reported to be performed in eight patients and all patient shows clinical improvement [21].

Ohshio et al. [7] employed a dorsal longitudinal incision to access the PIP joint. They accurately identified and forcefully detached the transverse retinacular ligaments from their attachment point on the palmar plate. The ligaments are flipped over onto the back side of the PIP joint to raise the lateral band towards the back and then stitched together. Following the surgery, the PIP joint is immobilized in an extended position using a K-wire for a period of four to five weeks. The method result in 4 out of 6 patients had complete recovery, while 2 others had extension contracture on the DIP due to disturbed palmar migration of the transverse retinacular ligament.

In our case, the first thing we did was central slip tenotomy, as we hope it would correct the extension mechanism of DIP. We actively ask the patient to extend the DIP but the goal was not achieved. Different with Curtis method, we did not start with tenolysis of terminal tendon as we believe central slip tenotomy and reconstruction would provide better extension mechanism. We then proceed using the Ohshio method to release the TRL and move the lateral slip dorsally and suture the TRL so the lateral slip would be on the dorsal side. In stage 2 of Curtis method, TRL was also divisioned, but in Ohshio's method, the TRL was flipped up and so the lateral band was also brought up and kept still dorsally, as illustrated in Fig. 4. We did this to achieve balance from the lateral slip to raise the force-extension on PIP joint to correct the abnormal flexion of the boutonniere deformity on PIP joint. After one year of follow-up, the patient was able to have full flexion-extension on the PIP and flexion on the DIP joint.

## 4 Conclusion

In summary, managing chronic boutonniere deformity presents significant challenges, especially when initial splinting and rehabilitation are delayed or ineffective. This case underscores the critical importance of timely intervention with splinting and rehabilitation in the acute phase to prevent permanent deformities. Ultimately, individualized treatment plans that consider the patient's specific injury and response to initial management are essential. Future studies should aim to establish more definitive guidelines to optimize the management of chronic boutonniere deformity, ensuring better functional outcomes and quality of life for patients. The Ala carte approach of reconstruction procedure using anesthesia yields good results. The importance of an intraoperative active range of movement evaluation plays a crucial role so that correction can be made accordingly.

## Consent

Written informed consent was obtained from the patients for publication and any accompanying images. A copy of the written consent is available for review by the Editor-in-Chief of this journal on request.

## Summary statement

No funding was received to assist with the preparation of this manuscript. All the data are available can be accessed via corresponding email after clearly stating the intention and permission to conduct research that requires our data. All authors in this research have given consent to participate in this manuscript and agree to this current form of manuscript to be submitted for publication.

## Ethical approval

Ethics approval is not required for case reports or case series deemed not to constitute research at your institution based on the regulation from our ethical committee, Health Research Ethics Committee FKUI-RSCM. In addition, Ethical approval was exempt at our institution since all patient's identity is blinded throughout this manuscript.

## Funding

The authors did not receive support from any organization for the submitted work. No funding was received to assist with the preparation of this manuscript. No funding was received for conducting this study.

## Author contribution

WL: study concept or design, data collection, data analysis and interpretation, writing the paper

RH: study concept or design, data collection, analysis and interpretation, writing the paper.

## Guarantor

WL:accepts full responsibility for the work and/or the conduct of the study, has access to the data, and controlled the decision to publish.

## Research registration number

Does not need any registration.

## Conflict of interest statement

The authors have no financial or proprietary interests in any material discussed in this manuscript, including any potential conflicts of interest regarding employment, consultancies, stock ownership, honoraria, paid expert testimony, patent applications/registrations, and grants or other funding.
